# Absent or atypical drainage of the ductus venosus

**DOI:** 10.1007/s00404-022-06828-2

**Published:** 2022-10-29

**Authors:** Karl Oliver Kagan, Rabih Chaoui, Markus Hoopmann

**Affiliations:** 1 Department of Obstetrics and Gynaecology, University of Tuebingen, Calwerstrasse 7, 72076 Tübingen, Germany; 2 Center for Prenatal Diagnosis and Human Genetics, Berlin, Germany

**Keywords:** Ductus venous, Liver, Prenatal, Genetic syndromes

The ductus venosus (DV) carries oxygen-rich blood from the umbilical vein (UV) to the heart, bypassing the fetal liver. In 1:500 to 1:2500 pregnancies, the DV is either missing or it drains at an atypical site. The types of DV abnormalities are grouped according to the drainage site (intra- or extrahepatic) and whether they bypass the liver (1,2). The presence of a DV abnormality increases the risk of other fetal defects, especially cardiac and genetic abnormalities (3).

In the image, color Doppler is used to demonstrate normal DV anatomy and five typical abnormalities:

Figure 1a, Normal DV anatomy. DV enters the inferior vena cava (IVC) close to the right atrium

**Fig. 1:**
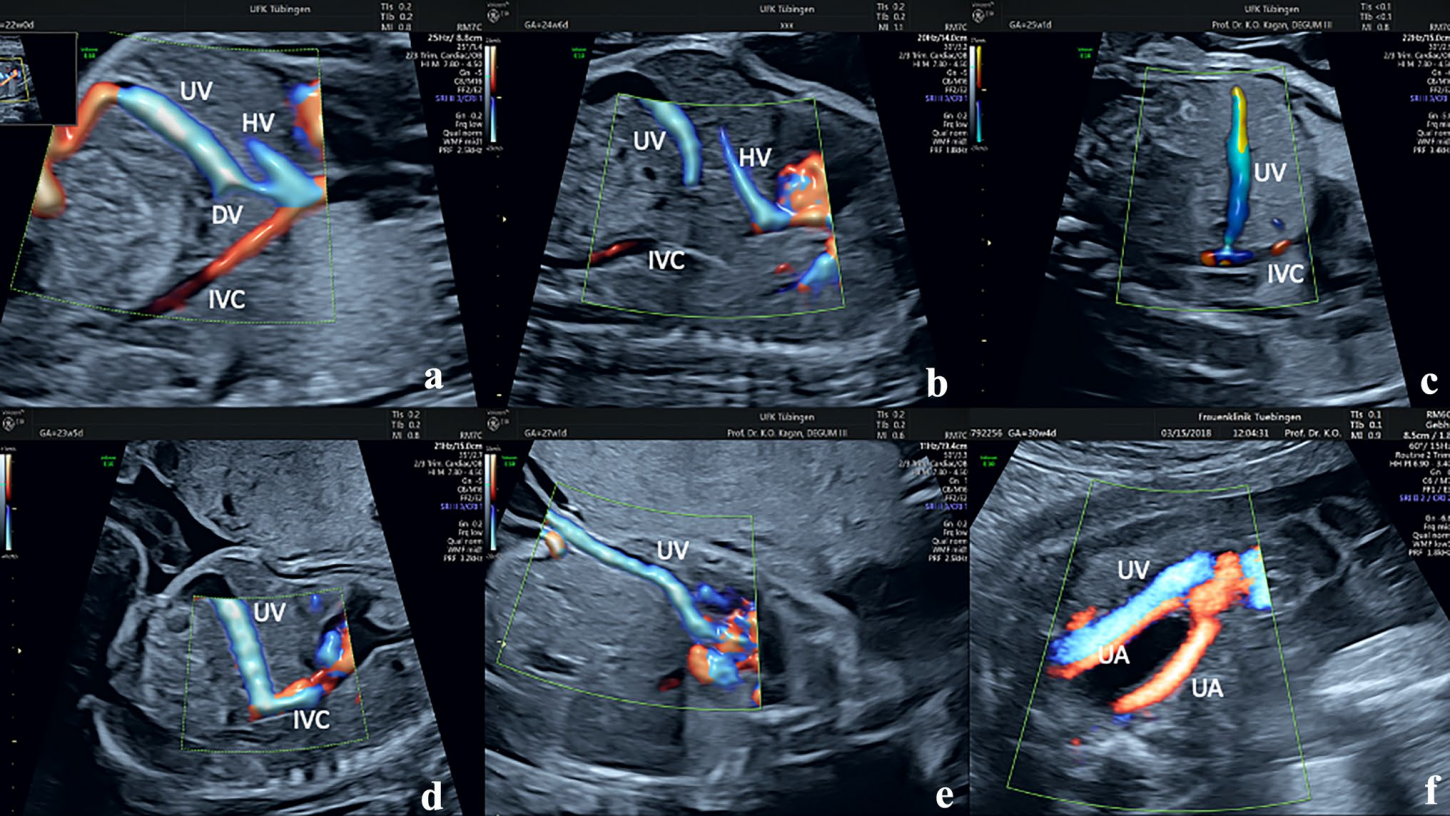
**a** Normal anatomy, **b-f** different types of absent DV and abnormal connection. DV: Ductus, UV: umbilical vein, IVC: inferior vena cava, HV: hepatic vein, UA: umbilical artery

Figure 1b, DV appears to be absent. The umbilical vein drains entirely into the liver without direct connection to the systemic venous circulation. This abnormality generally has a good outcome

Figure 1c, UV drains directly into the IVC at the level of the liver. A focal narrowing of the UV close to its entry into the IVC is present which causes “aliasing” in the Doppler studies. If the narrowing is present, the development of the portal venous system is generally normal

Figure 1d, This abnormality is similar to that in fig. 1c. However, the narrowing is absent. This allows for the blood to enter the IVC easily bypassing the liver. This results in underperfusion and underdevelopment of the portal venous system

Figure 1e, The UV courses over the anterior surface of the liver and enters the right atrium directly without an intervening DV

Figure 1f, The UV is located at the level of the urinary bladder and enters the systemic venous circulation either in the IVC or in the iliac vein

Figure 1e and 1f represents extrahepatic courses of the UV. The risk for other structural or genetic defects is high.

## References

[1] Achiron R, Kivilevitch Z (2016). Fetal umbilical–portal–systemic venous shunt: in-utero classification and clinical significance. Ultrasound Obst Gyn.

[2] Chaoui R, Heling K, Karl K (2014). Ultrasound of the Fetal Veins Part 1: the intrahepatic venous system. Ultraschall Med.

[3] Strizek B, Zamprakou A, Gottschalk I (2017). Prenatal diagnosis of agenesis of ductus venosus: a retrospective study of anatomic variants, associated anomalies and impact on postnatal outcome. Ultraschall Med.

